# Scleral thickness in patients with obstructive sleep apnea syndrome

**DOI:** 10.1007/s10384-025-01234-y

**Published:** 2025-06-21

**Authors:** Zeynep Akgun, Cumali Degirmenci, Sezai Tasbakan, Ozen K Basoglu, Melis Palamar

**Affiliations:** 1 Department of Ophthalmology, Faculty of Medicine, Ege University, 35040 Bornova, Izmir Turkey; 2 Department of Chest Diseases, Faculty of Medicine, Ege University, Izmir, Turkey

**Keywords:** Anterior segment optic coherence tomography, Obstructive sleep apnea syndrome, Scleral thickness

## Abstract

**Purpose:**

Evaluation of scleral thickness with anterior segment optical coherence tomography (AS-OCT) in patients with obstructive sleep apnea syndrome (OSAS).

**Study design:**

Cross-sectional study.

**Methods:**

A total of 30 severe (Group 1), 30 moderate (Group 2) and 30 mild (Group 3) OSAS patients and 22 healthy volunteers (Group 4) were included in the study. Scleral thickness measurements were taken with AS-OCT, 6 mm, 4 mm and 2 mm posterior to the scleral spur, in four gaze positions. Data and findings were examined comparatively**.**

**Results:**

At 6 mm posterior to the scleral spur, the mean thickness was found to be significantly higher in all quadrants in Group 1 (p<0.05 for all). Superior thickness was higher in Group 2 compared to Group 4 (p=0.034). At 4 mm posterior to the scleral spur; in Group 1, the mean thickness was found higher than Group 2 and 3 only in the inferior (p=0.01, p=0.021, respectively) and was found higher than Group 4 in the nasal and inferior (p<0.001, p=0.006, respectively). At 2 mm posterior to the scleral spur; significant difference was observed between Groups 1 and 2 only in the inferior (p=0.002), between Groups 1 and 3 in the nasal and inferior (p=0.029, p=0.002), and between Groups 1 and 4 the in nasal and inferior (p=0.002, p<0.001).

**Conclusion:**

The scleral thickness measured from 6 mm posterior to the scleral spur was higher in all quadrants in patients with severe OSAS. It is possible that the increase in scleral thickness, especially in severe OSAS, is due to extracellular matrix accumulation in the scleral tissue.

## Introduction

Obstructive sleep apnea syndrome (OSAS) is a chronic disease characterized by recurrent apnea and hypopnea attacks due to complete or partial obstruction of the upper airways during sleep [1]. OSAS is a common disease affecting 3.9% of women and 8.8% of men aged 30-70 years [2]. Diagnosis is made by polysomnography and the disease is classified as mild, moderate, and severe based on the Apnea-Hypopnea index (AHI), determined according to the number of complete/partial respiratory arrest attacks that occur during the sleep period. AHI:5-15 is grouped as mild, AHI: 15-30 as moderate, and AHI >30 as severe OSAS [3, 4].

OSAS patients frequently apply to sleep centers with complaints of loud and chronic snoring, attacks of shortness of breath during sleep, and daytime sleepiness. Among the ophthalmological pathologies associated with OSAS, floppy eyelid syndrome (FES), glaucoma, dry eye, and non-arteritic ischemic optic neuropathy are frequently encountered [5–7].

Optical coherence tomography (OCT) is a non-invasive imaging method that allows two-dimensional, high-resolution imaging of ocular tissues. Anterior segment optical coherence tomography (AS-OCT), which has been used frequently in the diagnosis and follow-up of ocular surface diseases, allows the visualization of corneal layers, anterior sclera, anterior chamber, the iris, and iridocorneal angle details [8].

Alterations in tissue structures and increase in the extracellular matrix due to chronic inflammation and hypoxia are involved in the pathophysiology of diseases associated with OSAS (e.g., FES and glaucoma) [9]. Any collagen-rich tissue such as the sclera may also be affected by similar mechanisms, potentially altering scleral thickness, and this may be one of the main mechanisms that constitute the pathophysiology of OSAS-related ocular diseases.

Considering this information, the present study aimed to evaluate scleral thickness via AS-OCT in patients with OSAS who had not yet started treatment and to compare the results with healthy volunteers.

## Materials and methods

Patients newly diagnosed with OSAS and no other accompanying systemic or ocular diseases, including central serous chorioretinopathy (CSR) along with healthy controls, were included in the study. OSAS classification was made according to AHI as mild, moderate or severe. Individuals with a spherical equivalent greater than ±0.5 D, those taking medication, using contact lenses, having a history of eye surgery, or exhibiting poor image quality (signal power density ≤41) were excluded from the study.

A detailed ophthalmological examination, including best-corrected visual acuity, anterior and posterior segment slit lamp examinations, and subsequently, scleral thickness measurements in 4 quadrants (superior, inferior, nasal, and temporal) were performed on all cases.

**Fig. 1:**
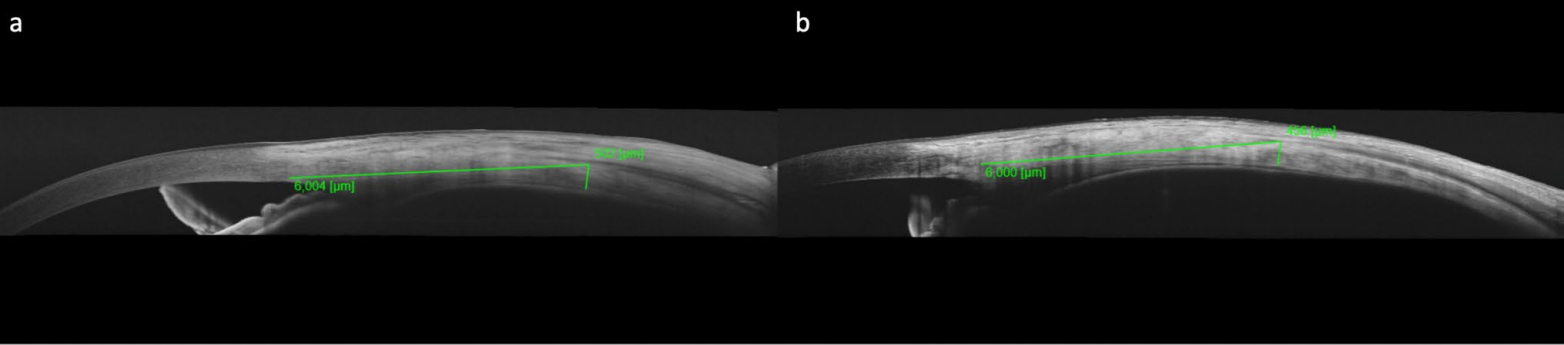
Anterior segment optical coherence tomography (AS-OCT) images of a severe Obstructive sleep apnea syndrome (OSAS) patient **(a)** and a healthy control **(b)** representing scleral thickness measurements from 6 mm

**Fig. 2:**
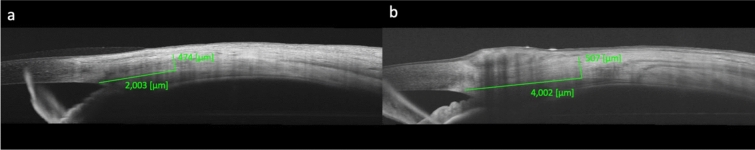
Scleral thickness measurements at 2 (a) and 4 (b) mm posterior to the scleral spur

Scleral thickness was measured with AS-OCT (Swept Source OCT Triton, Topcon) with the aid of an anterior segment lens compatible with images taken 6 mm posterior to the scleral spur, as described by Imanaga et al. [10], the anterior scleral border was determined by the difference between the rectus muscle with low reflectivity and the sclera with high reflectivity, while the posterior scleral border was defined by the signal originating from the choroid. Scleral thickness was measured vertically as the distance between the two borders (Fig. 1a, b). Additionally, measurements were obtained 2 mm and 4 mm posterior to the scleral spur in four quadrants to determine whether scleral thickness varied across different regions. In these assessments, the posterior border of the sclera was defined similarly, while the anterior border was considered to be the hyporeflective signal from the conjunctiva-Tenon's capsule and deep episcleral vessels (Fig. 2a, b). Participants were instructed to look in the respective direction to obtain images from the 4 quadrants of the sclera during imaging. All measurements were performed masked by the same clinician (Z.A.) at 8.00–9.00 a.m. to eliminate diurnal variations of anterior segment parameters and bias. AS-OCT scans were repeated three times for each patient, and the best-quality image was selected for analysis. To evaluate and eliminate variations, all scleral thicknesses were measured twice, approximately 10 days apart, using the same image by two blinded clinicians (Z.A., C.D.). The two measurements from each clinician and the mean values of the clinicians were compared. The intraclass correlation coefficient (ICC) for intra- and inter-observer reliability was found to be >0.80 for all parameters.

The data and findings were analyzed among all groups and in binary combinations such as Group 1 vs 2, Group 1 vs 3, etc. Additionally, the correlation between body mass index (BMI), which is one factor in the polysomnography data, and scleral thickness was examined.

Statistical analysis was performed using IBM SPSS Statistics 25.0 (IBM SPSS Statistics for Windows, Version 25.0.) package program. Numerical data were summarized with mean, standard deviation, median, minimum, and maximum values. The chi-square test was used for the analysis of gender. In pairwise comparisons, the T-test was used for parametric variables, the Mann-Whitney U test was used for nonparametric variables, and in comparisons involving three or more variables, ANOVA was used for parametric variables and the Kruskal-Wallis-H test was used for nonparametric variables. Post-hoc analyses were adjusted with Bonferroni correction. Pearson Correlation Analysis for parametric variables and Spearman Correlation Analysis for non-parametric variables were performed. The intra and inter observer reliability was assessed by the ICC. A value of p < 0.05 was considered as. statistically significant. prospective, cross-sectional study was conducted in accordance with the Principles of the Declaration of Helsinki, with the approval of the Ege University Faculty of Medicine Ethics Committee. Written informed consent was obtained from all participants.

## Results

Both eyes of 30 severe (Group 1), 30 moderate (Group 2), and 30 mild (Group 3) OSAS patients and 22 healthy volunteers (Group 4) were included in the study. The mean ages were 51.97±12.18 (24-77) in Group 1, 51.51±10.13 (28-71) in Group 2, 44.92±15.99 (20-74) in Group 3 and 46.42±11.80 (18-69) in Group 4 (p=0.070). Male-female ratios were 23/7 in Group 1, 24/6 in Group 2, 22/8 in Group 3 and 11/11 in Group 4, and male predominance was detected in all stages of OSAS compared to healthy volunteers (p=0.044).

The mean scleral thicknesses measured from 6 mm, 4 mm, and 2 mm posterior to the scleral spur are summarized in Table 1. Among all groups, a significant difference was detected in all quadrants in measurements taken from 6 mm posterior to the scleral spur, and in the nasal and inferior quadrants in measurements taken from 4- and 2-mm posterior to the scleral spur.

**Table 1:** The mean scleral thicknesses from 6 mm, 4 mm, and 2 mm posterior to the scleral spur in all quadrants

Scleral thickness (µm)		Group 1, n=60	Group 2, n=60	Group 3, n=60	Group 4, n=44	*p* values
-6 mm	*Nasal*	512.69 ±43.75	460.95±42.02	457.46±35.16	447.78±34.32	**<0.0001**
	*Temporal*	512.91±42.68	457.26±44.65	442.46±35.16	451.01±46.56	**<0.0001**
	*Superior*	505.44±46.17	458.68±44.14	449.96±52.89	439.57±42.21	**<0.0001**
	*Inferior*	518.55±35.49	467.36±38.02	470.07±33.66	460.66±33.92	**<0.0001**
-4 mm	*Nasal*	518.85±51.80	495.95±60.14	498.23±55.54	483.28±38.38	**0.014**
	*Temporal*	514.87±52.04	511.54±54.50	491.60±58.89	497.33±52.51	0.172
	*Superior*	486.72±53.66	481.40±57.35	464.76±57.44	471.64±47.33	0.285
	*İnferior*	522.40±52.25	490.75±50.95	479.93±73.04	497.47±41.70	**0.007**
-2mm	*Nasal*	530.25±37.95	516.13±51.45	502.76±58.31	507.83±36.36	**0.033**
	*Temporal*	518.69±55.35	521.69±58.33	517.26±58.87	515.45±47.62	0.824
	*Superior*	506.58±50.68	510.45±41.46	490.50±67.39	490.35±38.97	0.083
	*Inferior*	539.69±28.74	505.15±57.51	500.22±60.96	517.09±33.79	**0.001**

When the measurements made 6 mm posterior to the scleral spur were examined in binary combinations, the mean scleral thickness in all quadrants in Group 1 was found to be significantly higher than in all other groups (p<<0.001 for all quadrants). Only superior thickness was significantly higher in Group 2 compared to Group 4 (p=0.034). No significant difference was detected between Group 2 and Group 3 (p>0.05, for all) and between Group 3 and Group 4 in any quadrant (p>0.05, for all) (Table 2).

**Table 2:** p values of pairwise comparison between groups 6 mm posterior to the scleral spur

	Quadrants	Group 1	Group 2	Group 3	Group 4
Group 1	*Nasal*		**<0.0001**	**<0.0001**	**<0.0001**
	*Temporal*	-	**<0.0001**	**<0.0001**	**<0.0001**
	*Superior*		**<0.0001**	**<0.0001**	**<0.0001**
	*Inferior*		**<0.0001**	**<0.0001**	**<0.0001**
Group 2	*Nasal*	**<0.0001**		0.751	0.332
	*Temporal*	**<0.0001**	-	0.063	0.727
	*Superior*	**<0.0001**		0.574	**0.034**
	*Inferior*	**<0.0001**		0.598	0.587
Group 3	*Nasal*	**<0.0001**	0.751		0.515
	*Temporal*	**<0.0001**	0.063	-	0.246
	*Superior*	**<0.0001**	0.574		0.104
	*Inferior*	**<0.0001**	0.598		0.314
Group 4	*Nasal*	**<0.0001**	0.332	0.515	
	*Temporal*	**<0.0001**	0.727	0.246	-
	*Superior*	**<0.0001**	**0.034**	0.104	
	*Inferior*	**<0.0001**	0.587	0.314	

In pairwise comparison between groups at 4 mm posterior to the scleral spur (Table 3); the mean scleral thickness was found to be significantly higher in Group 1 in the inferior quadrant than in all other groups (p=0.001, p=0.021, p=0.006, respectively). Nevertheless, there was no difference between Groups 2, 3, and 4, in any quadrant (p>0.05 for all).

**Table 3:** *p* values of pairwise comparison between groups 4 mm posterior to the scleral spur

	Quadrants	Group 1	Group 2	Group 3	Group 4
Group 1	*Nasal*		0.185	0.101	**<0.0001**
	*Temporal*	-	0.961	0.144	0.067
	*Superior*		0.507	0.092	0.144
	*Inferior*		**0.001**	**0.021**	**0.006**
Group 2	*Nasal*	0.185		0.934	0.479
	*Temporal*	0.961	-	0.145	0.133
	*Superior*	0.507		0.198	0.471
	*Inferior*	**0.001**		0.737	0.983
Group 3	*Nasal*	0.101	0.934		0.101
	*Temporal*	0.144	0.145	-	0.710
	*Superior*	0.092	0.198		0.702
	*Inferior*	**0.021**	0.737		0.421
Group 4	*Nasal*	**<0.0001**	0.479	0.101	
	*Temporal*	0.067	0.133	0.710	-
	*Superior*	0.144	0.471	0.702	
	*Inferior*	**0.006**	0.983	0.421	

At 2 mm posterior to the scleral spur; a significant difference was observed between Group 1 and the others, especially in the inferior quadrant (Table 4). The superior quadrant was found to be significantly higher in Group 2 compared to Group 4 (p=0.008).

**Table 4:** *p* values of pairwise comparison between groups 2 mm posterior to the scleral spur

	Quadrants	Group 1	Group 2	Group 3	Group 4
Group 1	*Nasal*		0.274	**0.029**	**0.002**
	*Temporal*	-	0.968	0.795	0.438
	*Superior*		0.675	0.281	0.135
	*Inferior*		**0.002**	**0.002**	**<0.0001**
Group 2	*Nasal*	0.274		0.236	0.352
	*Temporal*	0.968	-	0.655	0.376
	*Superior*	0.675		0.169	**0.008**
	*Inferior*	**0.002**		0.584	0.649
Group 3	*Nasal*	**0.029**	0.236		0.668
	*Temporal*	0.795	0.655	-	0.778
	*Superior*	0.281	0.169		0.973
	*Inferior*	**0.002**	0.584		0.226
Group 4	*Nasal*	**0.002**	0.352	0.668	
	*Temporal*	0.438	0.376	0.778	-
	*Superior*	0.135	**0.008**	0.973	
	*Inferior*	**<0.0001**	0.649	0.226	

When the patients' BMIs were evaluated, there was no statistically significant difference between Group 1 and Group 2 (p=0.729), but the difference was significant between Groups 2- 4 (p=0.019). No correlation was found between the BMI and 4 quadrant scleral thickness (temporal, nasal, superior, inferior: r=0.260, 0.137, 0.039, 0.037, respectively).

## Discussion

Recent advances in ocular imaging and the development of portable anterior segment lenses compatible with OCT have made scleral thickness measurement a prevalent research topic in various anterior and posterior segment pathologies. While scleral thickness is influenced by various diseases, it remains unclear whether changes in scleral thickness are a primary factor in the pathogenesis or a secondary result of the underlying conditions in most cases [11, 12].

OSAS is associated with an increased risk of various sight-threatening or non-sight-threatening ocular disorders such as senile cataract, glaucoma, thyroid eye disease, FES, CSR, non-arteritic anterior ischemic optic neuropathy, conjunctival hyperemia, and dry eye syndrome [6]. OSAS has been associated with ocular diseases through three main mechanisms: Intermittent hypoxia and oxidative stress, elevated systemic inflammatory mediators [interleukin-6 (IL-6), IL-8, tumor necrosis factor-alpha (TNF-α), C-Reactive protein (CRP), matrix metalloproteinase-9 (MMP-9), vascular cell adhesion molecule (VCAM), intercellular adhesion molecule (ICAM), selectins] and overactivity of the sympathetic system [13–15].

When the relationship between OSAS and glaucoma is examined, it is seen that in addition to the damage to the optic nerve head, connective tissue changes that occur in the trabecular meshwork and the Schlemm canal play an important role in the pathophysiology. Both inflammation and oxidative stress damage these structures and disrupt drainage of aqueous humor. Gottanka et al. report increased extracellular matrix deposition and disruption of the actin cytoskeleton in the trabecular meshwork exposed to high levels of transforming growth factor (TGF)-β2 [5, 9, 9, 16]. In various clinical studies, it is claimed that changes in scleral thickness result in alterations to the configuration of the trabecular meshwork and Schlemm's canal, thereby playing a role in the pathogenesis of glaucoma. These studies found that scleral thickness in affected individuals was thinner or similar compared to healthy volunteers [17–19]. Although this contrasts with the increased scleral thickness observed in OSAS, scleral thickness may be an important factor in the pathophysiology of glaucoma development in OSAS. Further detailed studies on this subject are essential.

Another ocular pathology associated with OSAS is keratoconus, and several possible mechanisms have been proposed for its pathophysiology [20]. The serum concentration of MMP-9, which plays an important role in the formation of keratoconus and has the ability to break down collagen and other extracellular matrix proteins, was found to be high in patients with OSAS [21]. Moreover, anterior scleral thickness in patients with keratoconus showed significant variations compared to healthy volunteers [22].

CSR is also one of the pathologies associated with OSAS. Various studies report that OSAS is encountered with a frequency of 22% to 61% in patients with CSR [23]. Meta-analysis studies confirm the relationship between OSAS and CSR [24, 25]. The fundamental mechanism of CSR remains unclear, with the prevailing view suggesting that increased choroidal permeability, elevated choroidal hydrostatic pressure, and heightened serum cortisol and catecholamine levels lead to dysfunction of the retinal pigmented epithelium [26]. Overactivation of the sympathetic system is regarded as the fundamental cause of disrupted choroidal homeostasis and, consequently, the aforementioned pathologies [27–29]. The presented results indicate that scleral thickness increases, particularly in patients with severe OSAS. In addition to the known mechanisms, this may contribute to increased choroidal hydrostatic pressure and the development of CSR [30]. However, more studies, including molecular and animal research, are required to provide a clearer understanding of the subject.

As known, there is a positive correlation between OSAS and FES [21, 31]. The eyelid laxity, dermatochalasis and eyelash ptosis were more prominent in patients with OSAS than patients with simple snoring [32]. In histopathological examination, especially in severe OSAS, increased elastin content in the eyelid connective tissue, increased matrix metalloproteinase activity, and as a result excess connective tissue accumulation in the lateral canthal tendon were observed [7, 33]. Although not primarily related, similar mechanisms may also lead to increased scleral thickness.

Recent molecular studies about the role of scleral hypoxia in myopia progression show that scleral remodeling is associated with hypoxia. Upregulation of HIF-2α secondary to hypoxia contributes to the degradation of collagen type I α1 by increasing the expression of matrix metallopeptidase 2. HIF-1 promotes collagen deposition by regulating the expression of collagen prolyl-4-hydroxylase 1 and 2 in fibroblasts. Hypoxia also stimulates collagen synthesis in keloid fibroblasts [34]. To the best of our knowledge this is the first study in the literature that evaluates scleral thickness and investigates its pathophysiology in patients with OSAS. Herein, scleral thickness measurements made from 6 mm posterior to the scleral spur were higher in patients with severe OSAS than moderate-mild OSAS and healthy volunteers. In scleral thickness measurements made from 4- and 2-mm posterior to the scleral spur, thickening was observed especially in the inferior quadrant in severe OSAS compared to the other groups. One point to be considered is the male predominance in OSAS groups. It is reported in the literature that the scleral thickness of males is higher than females [28]. Nevertheless, when the scleral thickness of patients with moderate-mild OSAS, which is male-predominant, was compared with the scleral thickness of healthy volunteers, which had equal gender distribution, there was no statistically significant difference except in the superior quadrant between moderate OSAS and healthy volunteers in measurements made 6 and 2 mm posterior to the scleral spur. This suggests that gender distribution does not adversely affect the results. Although BMI values were found to be higher in severe OSAS, no correlation was found between BMI and scleral thickness. While there is no study that directly evaluates scleral thickness in obese patients, or patients with a high BMI, increased inflammation in these patients may cause extracellular matrix accumulation and elevated scleral thickness. However, the lack of correlation in our study group leads us to think that the increase in scleral thickness is related to the OSAS stage.

The main limitations of the presented study are that all measurements were made by a single operator and there is no well-defined standard technique for measuring scleral thickness. Furthermore, supporting the results with molecular and animal experimental studies would increase their reliability.

In conclusion, it is possible that the increase in scleral thickness, especially in severe OSAS, is due to extracellular matrix accumulation in the scleral tissue, with a mechanism similar to the chronic inflammation and oxidative stress damage observed in OSAS-related glaucoma and FES. Furthermore, increased scleral thickness may play a role in the pathophysiology of eye diseases in OSAS. Control of hypoxia and thus inhibition of scleral thickness increase may prevent the development of ophthalmic sequelae. Future studies, especially at the molecular level, will shed light on this issue.
